# Effect of the preoperative assessment of the anteroposterior diameters of the spinal canal and dural area on the efficacy of oblique lumbar interbody fusion in patients with lumbar spinal stenosis

**DOI:** 10.1186/s13018-023-03913-3

**Published:** 2023-06-19

**Authors:** Zhe Lu, Aoran Ding, Qingsong Yu, Haidong Wang, Lei Ma

**Affiliations:** Hebei Medical University, Shijiazhuang, China

**Keywords:** OLIF, Postoperative effect, Preoperative assessment, Dural area, Lumbar spinal stenosis, Anteroposterior diameter

## Abstract

**Objective:**

The purpose of this study was to quantify the degree of lumbar spinal stenosis by assessing the anterior and posterior vertebral canal diameter and dural area, determine the sensitivity of the anterior and posterior spinal canal diameter, dural area and dural occupying rate in predicting the postoperative efficacy of oblique lumbar interbody fusion (OLIF) for patients with single-stage lumbar spinal stenosis, and identify the corresponding indicators suggesting that OLIF surgery should not be performed.

**Methods:**

In a retrospective analysis of patients who had previously undergone OLIF surgery in our hospital, we included a total of 104 patients with lumbar spinal stenosis who had previously undergone single-stage surgery in our hospital. Three independent observers were employed to measure the anterior and posterior diameter of the spinal canal (AD, mm), dural area (CSA, mm^2^), the spinal canal area (SCA, mm^2^), and the ratio of the dural area to the spinal canal area (DM, %) at the disc level with the most severe stenosis on MRI. According to the values of AD and CSA in preoperative MRI, patients were divided into three groups: A, B, and C (Group A: AD > 12 and 100 < CSA ≤ 130, group B: Except A and C, group C: AD ≤ 10 and CSA ≤ 75). Preoperative and postoperative clinical outcome scores (Japanese Orthopaedic Association [JOA] score, VAS score, modified Macnab standard) of 104 patients were statistically.

**Results:**

There were significant differences in the preoperative and postoperative clinical correlation scores among the mild, moderate and severe lumbar spinal stenosis groups. The improvement rate of the post treatment JOA score, the difference between the preoperative and postoperative VAS score, and the modified Macnab standard were compared pairwise. There was no statistical significance in the improvement rate of the post treatment JOA score, the difference between the preoperative and postoperative VAS score, and the modified Macnab standard between Group A and Group B (*P* = 0.125, *P* = 0.620, *P* = 0.803). There were statistically significant differences between Group A and Group C and between Group B and Group C in the improvement rate of the JOA score, the difference in the pre- and postoperative VAS score, and the modified Macnab standard. The anterior and posterior vertebral canal diameter and dural area are sensitive predictors of the postoperative efficacy of OLIF surgery for single-stage lumbar spinal stenosis. Moreover, when the anterior and posterior vertebral canal diameter was less than 6.545 mm and the dural area was less than 34.43 mm^2^, the postoperative effect of OLIF surgery was poor.

**Conclusions:**

All the patients with mild, moderate, and severe lumbar spinal stenosis achieved curative effects after OLIF surgery. Patients with mild and moderate lumbar spinal stenosis had better curative effects, and there was no significant difference between them, while patients with severe lumbar spinal stenosis had poor curative effects. Both the anteroposterior diameter of the spinal canal and the dural area of the spinal canal were sensitive in predicting the curative effect of OLIF surgery for single-stage lumbar spinal stenosis. When the anterior and posterior vertebral canal diameter was less than 6.545 mm and the dural area was less than 34.43 mm^2^, the postoperative effect of OLIF surgery was poor.

## Introduction

Lumbar spinal stenosis (LSS) is a degenerative disease that can occur in bone, disc, or ligament structures [1]. Surgical treatment is one of the most effective methods for degenerative lumbar spinal stenosis, and most studies have shown that surgical treatment is superior to conservative treatment in the short and long term. Weinstein and other scholars have shown that surgical treatment of lumbar spinal stenosis is significantly better than conservative treatment. To reduce the risk of complications associated with traditional open surgery, surgeons have developed minimally invasive techniques such as oblique anterior lumbar interbody fusion, which allows direct entry into the lesion space through the muscle space to reveal the disc, achieve a more complete discectomy and a better fusion effect, and is becoming widely accepted [2–7].

OLIF allows entry through the physiological space between the retroperitoneal abdominal vascular sheath and the anterior edge of the psoas major muscle to locate the affected intervertebral disc. After excision, indirect decompression and fusion were performed with a fusion vessel inserted into the responsible intervertebral space. The operation was performed according to the methods introduced by Sato et al. [8]. Electrophysiological monitoring was not required during the operation. In OLIF, the right lateral position was accessed via the left approach, and the inferior vena cava was mostly located in the right front of the vertebral body. A skin incision of 3-4 cm was made along the external oblique muscle of the abdomen 5-8 cm in front of the midline, and the fibres of the external oblique muscle, internal oblique muscle and transverse muscle of the abdomen were separated by rigid dissection, allowing entry into the retroperitoneal space. The psoas muscles were pulled to the posterolateral muscle, and then a retractor was used to expose the intervertebral disc. After the intervertebral space was clearly exposed, the lateral fibrous ring was cut with a sharp knife. The intervertebral space and endplate were exposed by using nucleus pulposus forceps, a scraping spoon, and endplate scraping, and then the test mould was applied. After filling the bone graft material with the appropriate type of fusion device, the intervertebral implant was placed. OLIF was completed when a large fusion vessel was placed laterally from the vertebral body, thus restoring the height of the intervertebral space with a large bone graft area. OLIF achieves indirect decompression by reducing ligament flexion in the intervertebral disc space and enlarging the nerve pore area. OLIF resulted in a 30.2% increase in the median dural sac cross-sectional area [9] and a 30.0% increase in the average nerve foramen area [10]. OLIF can not only reduce the risk of destruction of the bone structure but can also reduce the exposure of the spinal canal, avoid pulling the nerve roots, and reduce the occurrences of cerebrospinal fluid leakage, nerve oedema and other related complications, and the long-term fusion rate is good. Qing-Yi Zhang analysed the early clinical efficacy of OLIF in the treatment of degenerative lumbar spine diseases, and then compared its efficacy with that of MIS-TLIF and found that OLIF has a shorter operation time, less intraoperative blood loss, and better leg pain relief, disc height recovery and anti-sagging ability [11].

OLIF is indicated for the treatment of mild to moderate lumbar spinal stenosis. It is generally believed that indirect decompression by OLIF is not effective in the treatment of severe lumbar spinal stenosis [12, 13]. However, Takayoshi Shimizu retrospectively analysed the postoperative effect of OLIF treatment on 42 patients with severe lumbar spinal stenosis and found in the follow-up process that the average expansion rate of the CSA (dural area) was 172.0% and 274.0% at 3 weeks and 1 year after surgery, respectively. OLIF is effective for some patients with severe lumbar spinal stenosis [14]. The relationship between the judgement of the degree of lumbar spinal stenosis and the efficacy of OLIF surgery is still unclear. The aim of this study is to determine the sensitivity of the anterior and posterior spinal diameter, dural area, and ratio of the dural area to the spinal area in evaluating the efficacy of OLIF in the treatment of lumbar spinal stenosis through a retrospective cohort analysis. Corresponding indicators were used to evaluate the efficacy of OLIF in the treatment of severe lumbar spinal stenosis.

## Methods

### General data and clinical grouping

This retrospective clinical study included 104 patients who underwent OLIF surgery for single-level lumbar spinal stenosis in our hospital from December 2013 to October 2022 (exclusion criteria were previous lumbar surgery (revision surgery), lumbar spondylolisthesis, noncentral lumbar disc herniation, infectious diseases and/or trauma, and spinal tumours).

No patients were lost to follow-up, as this retrospective study evaluated preoperative and predischarge outcome scores. A total of 104 patients (100%) were enrolled in the cohort, and their age, sex, body mass index, preoperative underlying diseases and symptom duration were recorded.

Magnetic resonance imaging (MRI) was performed for all the patients, and the anterior and posterior vertebral canal diameter (AD), dural area (CSA), and dural space occupancy rate (DM = dural area/spinal canal area) were measured by corresponding calculation and mapping software.

Criteria for the use of MRI: all MRI data were obtained through the 1.5 T system (Avanto SIEMENS). All patients received a lumbar MRI scan. The imaging protocol included the following: sagittal FRFSE T2WI sequence (TR 3500 ms and TE 120 ms), slice thickness of 4 mm, field of view of 11 scanning layers, matrix of 384 × 288.

A sagittal canal size less than or equal to 10 mm was considered absolute stenosis, while a sagittal canal size between 10 and 12 mm was considered relative stenosis [14]. The transverse surface area of the spinal canal or dural sac (DSCA) was measured in horizontal axial MRI sequences of the intervertebral disc. Surfaces less than 100 mm^2^ or 75 mm^2^ represent relative and absolute stenosis, respectively, and surfaces less than 130 mm^2^ represent early stenosis [15, 16] and according to the measured results, patients were divided into A (mild lumbar spinal stenosis), B (moderate lumbar spinal stenosis), and C (severe lumbar spinal stenosis) (Table 1).

**Table 1:** Grouping criteria for group A, Group B and Group C

	Group A	Group B	Group C
Criteria	AD > 12 and 100 < CSA ≤ 130	Except A and C	AD ≤ 10 and CSA ≤ 75
Total	18	58	28

AD (mm): Anterior and posterior vertebral canal diameter, CSA (mm^2^): dural area

We analysed the preoperative anterior and posterior vertebral canal diameter, dural area, and dural space occupying rate of the ineffective and effective patients after surgery and compared the sensitivity of these three indices in predicting postoperative effects before surgery.

### Measurement method

1. Anterior and posterior vertebral canal diameter (AD) measurement: Coronal MRI images of the lumbar disc with the most stenosis were taken, and the lines of the midpoint of the anterior vertebral canal (point a) and the midpoint of the posterior vertebral canal (point b) were taken, measure once per person, and the mean value was taken.
2. Dural area (CSA): Coronal MRI images of the lumbar intervertebral disc with the most stenosis were taken, marked around the outer dural margin, and the dural contour was formed by marking 20 points. The results were measure once per person, and the mean value was taken.
3. Measurement of dural space occupancy rate (DM): ① the spinal canal cross-sectional area (SCA) was determined by measuring the dural area.② the formula of DM is DM = CSA/SCA × 100%.

For the AD, the ICC across the 91 cases for the three observers was 0.925 (95% CI 0.889, 0.945). For CSA, the ICC was 0.917 (95% CI 0.875, 0.947). For SCA, the ICC was 0.902 (95% CI 0.867, 0.918).

(4) The highest overall JOA score is 29, and the lowest score is 0. A lower score indicates more dysfunction. Improvement rate of score after treatment = [(score after treatment – score before treatment)/29- score before treatment] × 100%. The improvement rate can be used to understand the clinical therapeutic effect. The improvement rate can also correspond to the commonly used efficacy criteria: cure is defined by an improvement rate of 100%, effective is defined by an improvement rate greater than 60%, effective is defined by an improvement of 25–60%, and ineffective is defined by an improvement of less than 25%.

The VAS score of the clinical evaluation was divided into "0–<3″(optimal), "3–<6″ (good), "6–≤8″ (can), and > "8″ (bad).

#### Macnab evaluation criteria

- Excellent: symptoms completely disappeared and the patient returned to original work and life activities;
- Good: patient still had mild symptoms, slightly limited activity and no impact on work and life;
- Fair: symptoms reduced, limited activity, limitations in performing normal work and life activities;
- Poor: no difference before and after treatment, even worse.

### Statistical analysis

SPSS 25.0 (IBM, Armonk, NY, USA) was used for analysis. Univariate analysis of variance was used to compare differences in age, symptom duration and muscle strength decline among the three groups; chi-square analysis was used to assess differences in sex and surgical segment among the three groups. Preoperative and postoperative clinical scores were compared among the three groups using one-way analysis of variance according to homogeneity of variance, and the comparison was conducted by a rank union test due to variances in preoperative and postoperative VAS scores in Group B. A P value < 0.05 was considered statistically significant.

## Result

A total of 104 patients, 36 males and 68 females, with an average age of 59.16 years (37-78 years), were included in the study. All patients were patients with single-stage lumbar spinal stenosis.

It can be seen from Table 2 that among the three groups, the number of patients undergoing OLIF surgery for moderate lumbar spinal stenosis was the largest, but there was no significant difference in the number of patients among the three groups, and the descriptive characteristics of patients with different degrees of stenosis were similar (*P* > 0.05). In terms of symptom duration and muscle strength reduction, there were statistically significant differences among patients with different degrees of stenosis (*P* < 0.05).

**Table 2:** Comparison of descriptive characteristics, preoperative symptoms and duration of symptoms in groups A, B and C

	A group	B group	C group	*P*
Sex				0.543
Male	8	20	8	
Female	10	38	20	
Age (years)	57.11 ± 6.694	59.29 ± 7.696	60.21 ± 9.402	0.437
Symptom duration (months)	3.44 ± 1.58	10.02 ± 2.632	15.89 ± 3.957	0.000
Decreased muscle strength	0	4	9	0.001

Table 3 shows the comparison of anterior and posterior spinal diameter (AD), dural area (CSA), dural space occupation rate (DM), and parameters of the operative segment in the three groups of patients. There were statistically significant differences in the anterior and posterior spinal diameter (AD) and the dural area (CSA) among the three groups (*P* < 0.001). There was no significant difference between the dural space occupancy rate (DM) or surgical level (P dural space occupancy rate =0.066, P surgical level =0.780).

**Table 3:** Comparison of anterior and posterior spinal diameter (AD), dural area (CSA), dural space occupancy rate (DM) and surgical parameters in groups A, B and C

	Group A	Group B	Group C	*P*
AD (mm)	13.67 ± 0.87	11.49 ± 1.17	8.29 ± 1.14	0.000
CSA (mm^2^)	116.77 ± 11.80	75.84 ± 18.32	36.89 ± 9.04	0.000
DM (%)	0.82 ± 0.05	0.78 ± 0.06	0.78 ± 0.05	0.066
Surgical segments				0.780
L3–L4	7	21	10	
L4–L5	11	27	18	

To determine whether the postoperative improvement of the patients was statistically significant, the preoperative JOA score, postoperative JOA score, preoperative VAS score and postoperative VAS score of the 3 groups were compared in pairs (Table 4). There were significant differences between the preoperative and postoperative JOA scores of patients in Groups A, B and C (*P* < 0.000, *P* < 0.000, *P* = 0.001). The difference between the preoperative VAS score and postoperative VAS score of the three groups was statistically significant (*P* < 0.000, *P* < 0.000, *P* = 0.017). It could be seen from the scores of the two groups that patients in the light, medium and severe groups did have effective improvement after surgery, but it could be seen from the difference in preoperative and postoperative scores of patients in the light, medium and severe groups. The postoperative score improvement in Group C was significantly lower than that in Groups A and B, and the degree of postoperative improvement in Group C was lower.

**Table 4:** Intra-group comparison of preoperative and postoperative JOA and VAS scores in groups A, B and C

	JOA			VAS		
	Pre-OP	Post-OP	*p*	Pre-OP	Post-OP	*p*
Group A	22.89 ± 1.682	25.26 ± 1.464	< 0.000	5.17 ± 1.917	2.39 ± 1.243	< 0.000
Group B	17.07 ± 2.034	21.79 ± 2.654	< 0.000	6.47 ± 0.922	3.86 ± 1.572	< 0.000
Group C	9.54 ± 2.136	12.21 ± 3.446	0.001	7.55 ± 1.266	6.68 ± 1.926	0.017

To clarify the difference in postoperative effect improvement between the three groups, the improvement rate of the post treatment JOA score, the difference in the pre- and postoperative VAS score, and the modified Macnab standard were compared in pairs (Table 5) in Groups A, B and C. There was no significant difference between Group A and Group B in the improvement rate of the post treatment JOA score, the pre- and postoperative VAS score, and the modified Macnab standard (*P* = 0.125, *P* = 0.620, *P* = 0.803). There were significant differences in the improvement rate of the JOA score, the pre- and postoperative VAS score and the modified Macnab standard between Group A and Group C (*P* < 0.000, *P* < 0.000, *P* = 0.003). There were significant differences in the improvement rate of the JOA score, the pre- and postoperative VAS score and the modified Macnab standard between Group B and Group C (*P* < 0.000, *P* < 0.000, *P* < 0.000).

**Table 5:** Comparison of postoperative score improvement in groups A, B and C

	Group A	Group B	*P*	Group A	Group C	*P*	Group B	Group C	*P*
JOA improvement	0.466 ± 0.168	0.403 ± 0.167	0.125	0.466 ± 0.168	0.144 ± 0.102	0.000	0.403 ± 0.167	0.144 ± 0.102	0.000
VAS improvement	2.78 ± 1.478	2.60 ± 1.350	0.620	2.78 ± 1.478	1.07 ± 1.052	0.000	2.60 ± 1.350	1.07 ± 1.052	0.000
Improved MacNab	2.22 ± 0.647	2.28 ± 0.812	0.803	2.22 ± 0.647	2.96 ± 0.838	0.000	2.28 ± 0.812	2.96 ± 0.838	0.000

JOA improvement = [(score after treatment – score before treatment)/29- score before treatment] × 100%

VAS improvement =|Postoperative VAS score-Preoperative VAS score|

From the above data, we can see that the postoperative efficacy of OLIF surgery for severe lumbar spinal stenosis is improved, but the degree of improvement is inferior to that of OLIF surgery for mild and moderate lumbar spinal stenosis. According to our statistics, of the 104 patients that were included, 20 had poor or even worsened symptoms after OLIF surgery. Among the 20 patients, 12 had severe lumbar spinal stenosis after surgery, and 8 had moderate lumbar spinal stenosis after surgery. The postoperative effect of mild lumbar spinal stenosis was generally good. We divided the 104 patients into ineffective and effective patients according to postoperative outcomes. We analysed the preoperative anterior and posterior vertebral canal diameter, dural area, and dural space occupying rate of the ineffective and effective patients after surgery and compared the sensitivity of these three indices in predicting postoperative effects before surgery.

**Fig. 1:**
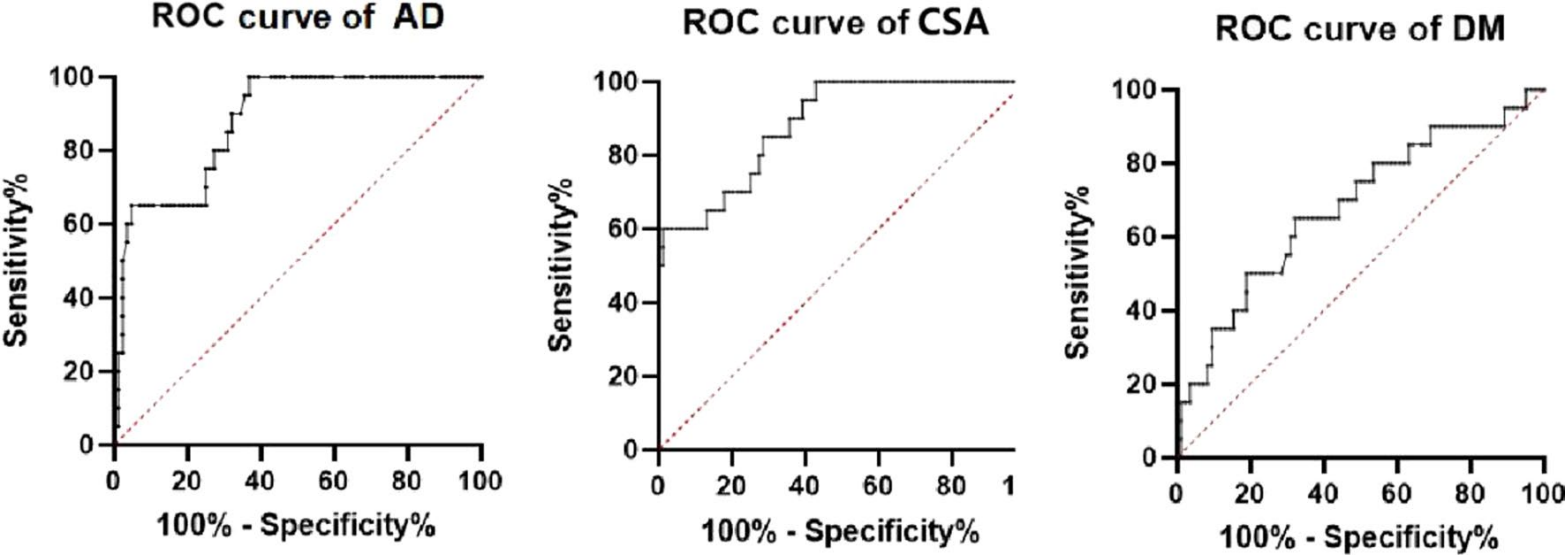
Sensitivity analysis and comparison of AD, CSA and DM for preoperative evaluation of postoperative effects of OLIF

The ROC curve was drawn by GraphPad Prism software, and the following three sets of graphs were obtained (Fig. 1).

The ROC curve of the anterior and posterior vertebral canal diameters showed that the anterior and posterior vertebral canal diameters had good value in evaluating the postoperative efficacy of OLIF surgery for lumbar spinal stenosis. (AUC = 0.8798, 95% CI 0.8064–0.9531, *P* < 0.0001). The ROC curve of the dural area showed that the dural area also had good value in the preoperative prediction of the postoperative efficacy of OLIF surgery in patients with lumbar spinal stenosis. (AUC = 0.8839, 95% CI 0.8083–0.9596, *P* < 0.0001). The ROC curve of the dural area occupying rate showed that the dural area occupying rate did not have good value in the preoperative prediction of the postoperative efficacy of OLIF surgery for lumbar spinal stenosis when compared with the value of the anterior and posterior vertebral diameter and the dural area (AUC = 0.6783, 95% CI 0.5417–0.8149, *P* < 0.0135).

Table 6 shows that dural area has the most sensitivity among the three groups of data in predicting the postoperative effect of OLIF for lumbar spinal stenosis. Through calculation software, it was calculated that the postoperative effect of OLIF surgery is not good for patients with lumbar spinal stenosis whose dural area is less than 34.43 mm^2^. The anterior and posterior spinal canal diameters are also sensitive in predicting the postoperative effect of OLIF for lumbar spinal stenosis. The postoperative effect of OLIF surgery is not good for lumbar spinal stenosis patients with an anterior and posterior spinal canal diameter less than 6.545 mm, and there is no significant difference in sensitivity between the two groups. However, the dural area occupying rate was much less sensitive than the anterior and posterior vertebral diameter and dural area in predicting the postoperative outcome of OLIF surgery in patients with lumbar spinal stenosis.

**Table 6:** Sensitivity and critical values of AD, CSA and DM for preoperative evaluation of postoperative effects of OLIF

	AD (mm)	CSA (mm^2^)	DM (%)
AUC	0.8798	0.8839	0.6783
Cut off	< 6.545	< 34.43	> 0.7660

## Discussion

OLIF surgery has a good effect on patients with mild and moderate lumbar spinal stenosis. OLIF has the advantage of posterior margin space height recovery. OLIF has more advantages in that it restores spinal balance and reduces the risk of certain diseases, such as long-term degeneration of adjacent segments [17, 18]. Goel proposed that lumbar spinal stenosis can be treated with arthrofusion alone, thus resolving pathological spinal instability [19, 20]. In addition, OLIF allows for more thorough disc removal, better indirect decompression, larger bone graft area, and allowing the placement of a larger fusion device in the epiphyseal ring, which is more conducive to postoperative intervertebral fusion. Therefore, it has been suggested that "indirect decompression" and "stabilization" play key roles in the treatment of lumbar spinal stenosis [21]. However, for severe lumbar spinal stenosis cases, most surgeons prefer direct decompression, arguing that OLIF surgery is generally not appropriate [12, 22]. However, MRI has been used to demonstrate that the capsule dilates over time. Even in the case of severe stenosis, the CSA increases over time, from 54.5 ± 19.2 mm^2^ before surgery to 84.7 ± 31.8 mm^2^ at 3 weeks after surgery and then to 132.6 ± 37.5 mm^2^ at the last follow-up [23]. In this context, this study quantified the degree of lumbar spinal stenosis by measuring the anterior and posterior spinal diameter and the dural area, determining the sensitivity of the anterior and posterior spinal diameter, dural area and dural space occupancy rate in predicting the postoperative efficacy of OLIF for patients with single-stage lumbar spinal stenosis, and identifying the corresponding indicators that OLIF surgery should not be performed. Our study found that patients with mild and moderate lumbar spinal stenosis received good results after OLIF, and there was no significant difference between them. However, OLIF was less effective after surgery in patients with severe lumbar spinal stenosis, which was different from the other two groups. And a greater proportion of patients with severe lumbar spinal stenosis had poor postoperative improvement. However, we still found that some patients in Group C with severe lumbar spinal stenosis had better results after OLIF. Therefore, we further investigated and found that dural area was the most sensitive of the three indicators for predicting the outcome of OLIF in patients with lumbar spinal stenosis (AUC = 0.8839). The sensitivity of anterior and posterior vertebral canal diameters (AUC = 0.8798) was similar to that of the dural area. Dural space occupancy was less sensitive to predict postoperative outcome (AUC = 0.6783). In addition, by calculation, we found that the postoperative effect of OLIF surgery was poor when the anterior and posterior vertebral canal diameters were less than 6.545 mm and 34.43 mm^2^, respectively, in patients with single-stage lumbar spinal stenosis.

This study also has limitations: (1) The sample size of patients with single-stage lumbar spinal stenosis was small. (2) This study only collected and counted clinical scores before discharge, at which time patients may still have some postoperative complications affecting the accuracy of scores. (3) In this study, only the anterior and posterior diameters of the spinal canal and the dural area were selected as indicators, which were divided into three groups: mild, moderate and severe. Thus, it is still necessary to further improve the grouping indicators and refine the grouping.

## Conclusion

In this study, patients with mild, moderate and severe lumbar spinal stenosis all achieved curative effects after OLIF surgery, patients with severe lumbar spinal stenosis had poor curative effects after OLIF surgery, and patients with mild and moderate lumbar spinal stenosis had good curative effects after OLIF surgery, and there was no significant difference between them. Both the anteroposterior diameters of the spinal canal and the dural area are sensitive in predicting the postoperative efficacy of OLIF surgery for single-stage lumbar spinal stenosis. When the anteroposterior spinal canal diameter is less than 6.545 mm and the dural area is less than 34.43 mm^2^, the postoperative efficacy of OLIF surgery is poor.

## Data Availability

The datasets used and/or analyzed during the current study are available from the corresponding author on reasonable request.
